# Molecular Detection of *Bartonella* spp. in China and St. Kitts

**DOI:** 10.1155/2019/3209013

**Published:** 2019-09-03

**Authors:** Ke Huang, Patrick John Kelly, Jilei Zhang, Yi Yang, Weiguo Liu, Anwar Kalalah, Chengming Wang

**Affiliations:** ^1^Yangzhou University College of Veterinary Medicine, Yangzhou, Jiangsu 225009, China; ^2^Ross University School of Veterinary Medicine, Basseterre, Saint Kitts and Nevis; ^3^Department of Medicine, University of Illinois at Chicago, Chicago, IL 60612, USA; ^4^Anhui Science and Technology University, Bengbu, Anhui, China; ^5^Auburn University College of Veterinary Medicine, Auburn, AL 36849, USA

## Abstract

*Bartonella* are vector-borne hemotropic bacteria that infect a wide variety of hosts, including people. While there are PCR assays that can identify individual or groups of *Bartonella*, there is no reliable molecular method to simultaneously detect all species while maintaining genus specificity and sensitivity. By comparing highly conserved *16S rRNA* sequences of the better-recognized *Bartonella* spp. on GenBank, we selected primers and probes for a genus-specific pan-*Bartonella* FRET-qPCR. Then, a *gltA*-based *Bartonella* PCR was established by selecting primers for a highly variable region of *gltA*, of which the sequenced amplicons could identify individual *Bartonella* spp. The pan-*Bartonella* FRET-qPCR did not detect negative controls (*Brucella* spp., *Anaplasma* spp., *Rickettsia* spp., *Coxiella burnetii*, and *Wolbachia*) but reliably detected as few as two copies of the positive control (*Bartonella henselae*) per reaction. There was complete agreement between the pan-*Bartonella* FRET-qPCR and the *gltA*-based *Bartonella* PCR in detecting *Bartonella* in convenience test samples from China and St. Kitts: cats (26%; 81/310), *Ctenocephalides felis* (20%; 12/60), cattle (24%; 23/98), and donkeys (4%; 1/20). Sequencing of the *gltA*-based *Bartonella* PCR products revealed *B. henselae* (70%; 57/81) and *B. clarridgeiae* (30%; 24/81) in cats and *C. felis* (67%; 8/12, and 33%; 4/12, respectively) and *B. bovis* in cattle (23.5%; 23/98) and donkeys (4.0%; 1/24). The pan-*Bartonella* FRET-qPCR and *gltA*-based *Bartonella* PCR we developed are highly sensitive and specific in detecting recognized *Bartonella* spp. in a single reaction. The pan-*Bartonella* FRET-qPCR is convenient requiring no gel electrophoresis and providing copy numbers, while the *gltA*-based *Bartonella* PCR reliably differentiates individual *Bartonella* species. The use of these PCRs should greatly facilitate large-scale surveillance studies and the diagnosis of infections in clinical samples.

## 1. Introduction

There are now over 40 species and subspecies of *Bartonella* which are small, intracellular, vector-borne hemotropic Gram-negative bacteria. High prevalences of the organisms have been reported around the world in a wide range of insect vectors and domestic and wild animal hosts including rodents, felines, canines, ruminants, and even bats [1–4]. In China, *Bartonella* species have been detected in a wide range of animals [5–8] with at least 10 *Bartonella* species having been implicated in human diseases that range from self-limiting regional lymphadenitis to severe endocarditis [9–11]. To the best of our knowledge, there has been only a single report of *Bartonella* infecting people in the Caribbean [12] although infections have been described in cats [13], mongooses [14], bats [15, 16], dogs [17, 18], rodents [19], cat and rodent fleas [20, 21], and bat flies [22].

Detecting *Bartonella* species in their vectors and mammalian hosts is often not easy as it is difficult and time-consuming to culture them and serology may be unreliable. Although numerous molecular methods to detect *Bartonella* have been described [17–28], in most cases, a single PCR only detects individual or closely related species of *Bartonella* [29]. Although multiplex PCRs have been described to detect *Bartonella*, they require a wide range of primers and probes to detect all species. Furthermore, the PCRs have poor sensitivity (with up to 8 × 10^7^ bacteria per g being undetectable in feces) [30, 31] because of inhibitors in environmental and clinical samples. Recently, a highly specific and quantitative qPCR has been established to specifically amplify *nuoG* of the *Bartonella* genus and identified *B. henselae* and *B. clarridgeiae* in cats in Brazil [32].

We used the available *16S rRNA* sequences of 20 *Bartonella* species in GenBank to develop and validate a highly sensitive and genus-specific pan-*Bartonella* FRET-qPCR. We also used available sequences for the citrate synthase gene (*gltA*) and considered a reliable tool for distinguishing genotypes [33], to develop a *gltA*-based *Bartonella* PCR to identify and differentiate the species. The development of these PCRs and their use on convenience samples from China and St. Kitts is described below.

## 2. Materials and Methods

### 2.1. Whole Blood Samples and Fleas

This study was reviewed and approved by the Institutional Animal Care and Use Committee of the Yangzhou University College of Veterinary Medicine, China (YZU-13-58Wang), and of the Ross University School of Veterinary Medicine, St. Kitts (07/02/3MOURA). Convenience whole blood samples in EDTA were collected from cats in China and St. Kitts. Between April 2013 and June 2014, 164 cat whole blood samples were collected from five cities in four provinces in China and stored at −80°C until DNA extraction (see below). The cats sampled in Yangzhou were kept in a shelter, while those from Beijing, Shanghai, and Guangdong were pet cats presenting to veterinary clinics with a variety of conditions. Cat fleas (*n* = 60) were collected from the cats used for blood sampling, and the flea species were identified according to the morphological criteria as described in previous studies [34, 35]. Frozen (−80°C) whole blood samples were also available from apparently healthy stray cats from St. Kitts (*n* = 146) sampled during a Trap, Neuter, Release program conducted in 2012. Archived whole blood samples from cattle (*n* = 98), sheep (*n* = 30), donkeys (*n* = 24), and horses (*n* = 24) collected as described in previous studies [35–38] were also used in this project. Storage and laboratory analysis of all the samples were performed in Yangzhou University, Yangzhou, China.

### 2.2. DNA Extraction

Samples were thawed at room temperature, and DNA was extracted from whole blood and homogenized arthropods [33] with the QIAamp® DNA Blood Mini Kit (Qiagen, Valencia, CA, USA), following the manufacturer's protocol. DNA was frozen at −80°C until PCRs were performed. The concentration and quality of the samples in this study were validated by running PCR endogenous control of the universal hydroxymethylbilane synthase-based PCR as described [39].

### 2.3. Development of a *16S rRNA*-Based Pan-*Bartonella* FRET-qPCR

#### 2.3.1. Primers and Probes

**Figure 1:**
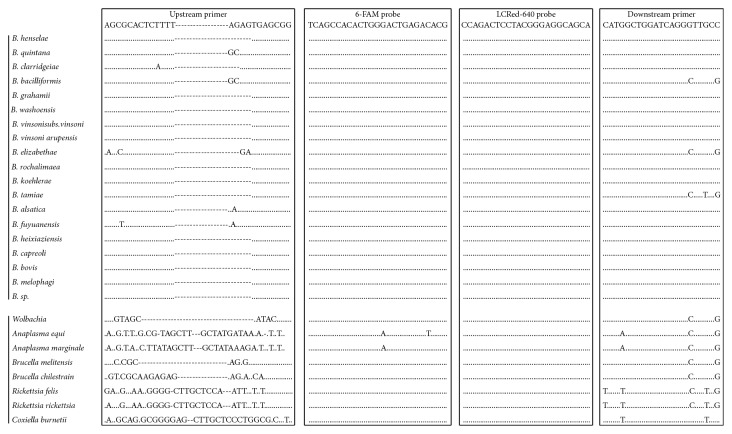
Alignment of oligonucleotides for the pan-*Bartonella* FRET-qPCR based on the *16S rRNA* used in this study. Primers and probes are shown at the top of the boxes. Dots indicate nucleotides identical to primers and probes, and dashes denote absence of the nucleotide. The upstream primers and two probes are used as the indicated sequences, while the downstream primer is used as the antisense oligonucleotide. The designed oligonucleotides show minimum mismatching with *Bartonella* spp. (0 mismatches with 14 species, 1 mismatch with 3 species, 2 mismatches with 1 species, and 4 mismatches with 2 species) but 11–29 nucleotide mismatches with the related non-*Bartonella* species. The 6-FAM label is directly attached to the 3-terminal nucleotide of the fluorescein probe, and the LCRed-640 fluorescein label is added via a linker to the 5′ end of the LCRed-640 probe.

The *16S rRNA* sequences for the 18 better-recognized *Bartonella* spp. on GenBank and eight closely related species were obtained from GenBank: *B. quintana* (NR_044748), *B. henselae* (AJ223778), *B. clarridgeiae* (NR_03696), *B. elizabethae* (AB246807), *B. bacilliformis* (NR_044743), *B. bovis* (NR_025121), *B. capreoli* (NR_025120), *B. koehlerae* (NR_024932), *B. alsatica* (NR_025272), *B. grahamii* (NR_029366), *B. vinsonii* subsp. *berkhoffii* (DQ2281315), *B. vinsonii* subsp. *vinsonii* (NR_037056), *B. vinsonii* subsp. *arupensis* (AF214558), *B. doshiae* (NR_029368), *B. queenslandensis* (EU111758), *B. washoensis* (AB519066), *B. rattaustraliani* (EU111753), *B. tamiae* (DQ395176), *B. rochalimae* (DQ683196), *B. melophagi* (AY724770), *Bartonella* sp. (U71322), *Anaplasma phagocytophilum* (AF172167), *Anaplasma marginale* (AF414873), *Wolbachia* (KF249715), *Brucella melitensis* (CP002932), *Brucella chilestrain* (AY513509), *Rickettsia felis* (NR_074483), *R. rickettsii* (L36217), and *Coxiella burnetii* (D89798) (Figure 1). These sequences were aligned using Clustal multiple sequence alignment in VectorNTI (Informax Inc., North Bethesda, MD, USA) to identify a highly conserved region of the *16S rRNA* common to all the above *Bartonella* spp., but significantly different from the other species (Figure 1). The primers and probes we developed were situated within the conserved region and synthesized by Integrated DNA Technologies (Coralville, IA, USA). The pan-*Bartonella* FRET-qPCR we established amplifies a 304 bp target with the positions of primers and probes shown in Figure 1: forward primer: 5′-AGCGCACTCTTTTAGAGTGAGCGG-3′; reverse primer: 5′-CATGGCTGGATCAGGGTTGCC-3′; anchor probe: 5′-TCAGCCACACTGGGACTGAGACACG-(6-FAM)-3′; and reporter probe: 5′-(LCRed640)-CCAGACTCCTACGGGAGGCAGCA-phosphate-3′.

#### 2.3.2. Thermal Cycling

The pan-*Bartonella* FRET-qPCR was performed in a LightCycler 480® II real-time PCR platform with 20 *μ*l final volumes containing 10 *μ*l extracted DNA. The primers and probes were 1 *μ*M and 0.2 *μ*M in the final reaction system, respectively. The qPCR buffer was established with a final concentration of 20 mM Tris-HCl, 4.5 mM MgCl_2_, 0.05% Tween 20, and 0.05% Nonidet P-40. Thermal cycling consisted of a 2 min denaturation step at 95°C followed by 18 high-stringency step-down thermal cycles and 40 low-stringency fluorescence acquisition cycles for 1 sec at 95°C, 12 sec at 58°C, 30 sec at 67°C, and 10 sec at 72°C. Melting curve analysis was performed by monitoring fluorescence between 38°C and 80°C. All the PCRs were run in duplicate in this study.

#### 2.3.3. Specificity

*Bartonella henselae* DNA from a cat in St. Kitts [21] was used as the positive control, and the PCR product was confirmed by electrophoresis (1.5% MetaPhor agarose gels) and by purification using QIAquick PCR Purification Kit (Qiagen, Valencia, CA, USA) and genomic sequencing (GenScript, Nanjing, Jiangsu, China). We used BLAST to compare the sequencing data from the positive *Bartonella* samples with the available *Bartonella* sequences in GenBank. The specificity of the PCR was further verified using the related species *Brucella melitensis*, *Brucella chilestrain*, *Wolbachia*, *Coxiella burnetii*, *Rickettsia felis*, *Rickettsia rickettsii*, *Anaplasma phagocytophilum*, and *Anaplasma marginale* (provided by the Parasitological Laboratory of Yangzhou University College of Veterinary Medicine).

#### 2.3.4. Sensitivity

For quantitative standards, we used amplified DNA of *B. henselae* identified in this study. The amplification products were gel purified using the QIAquick Gel Extraction Kit (Qiagen, Valencia, CA, USA) and quantified using the PicoGreen DNA fluorescence assay (Molecular Probes, Eugene, OR, USA). The molarity of the DNA was estimated using the calculated molecular mass of the amplicons and dilutions made to give solutions containing 10,000, 1,000, 100, 10, and 1 gene copies/*μ*l in T_10_E_0.1_ buffer. These were used as quantitative standards in the FRET-qPCR surveys to enable standard curves to be developed for the calculation of the gene copy numbers in positive samples. Twofold dilutions of 10 and 1 gene copies/*μ*l solution were used to determine the minimal detection limit.

### 2.4. Development of a Citrate Synthase Gene- (*gltA*-) Based PCR to Differentiate *Bartonella* spp

**Figure 2:**
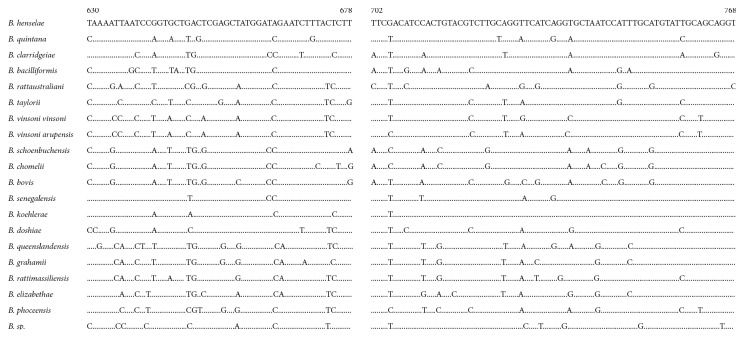
Alignment of oligonucleotides for *Bartonella* PCR based on *gltA* used in this study. Two partial amplicons of polymorphic regions of *Bartonella* spp. based on *gltA* are shown. The base of *B. henselae* (L38987.1) shown at the top of the figure was complete with the coding sequences from 630 to 678 and 702 to 768. Dots indicate identical nucleotide sequence to that of *B. henselae*. Through the polymorphic region of *Bartonella* spp., different *Bartonella* species could be determined by genomic sequencing in this study. The number of nucleotide mismatches between *B. henselae* and other *Bartonella* species in the 116 polymorphic coding sequences is also noted (mismatch in coding sequences from 630 to 678 and 702 to 768 with about 5 and 23 nucleotides). In this polymorphic region, none of *Babesia* species had identical sequences.

The amplicon of the pan-*Bartonella* FRET-qPCR we established was based on a region of the *16S rRNA* that is highly conserved among different *Bartonella* spp. and is thus ideal for screening samples for the organisms. To differentiate *Bartonella* spp. that gave positive reactions in screening pan-*Bartonella* FRET-qPCR, we used a standard PCR that amplifies a relatively highly polymorphic region of the citrate synthase gene (*gltA*) (439 nucleotides for different *Bartonella* spp.) and sequenced the products (GenScript, Nanjing, Jiangsu, China). For the *gltA*-based *Bartonella* PCR, we designed, as above for the pan-*Bartonella* FRET-qPCR, a forward primer (5′-CGTAATGATCTYAGTTAYGCTGCAAA-3′) and a reverse primer (5′-AGAAGTGGATCATTTTGAATRTTBARYTC-3′) that amplify all the 18 better-recognized *Bartonella* spp. available in GenBank (Figure 2). The sensitivity and specificity of the gltA-based PCR were assessed as described above for the pan-*Bartonella* FRET-qPCR. The PCRs were performed in a LightCycler 480® II real-time PCR platform with 20 *μ*l final volumes containing 10 *μ*l extracted DNA, and 1 *μ*M primers with the same PCR buffer as described above. Thermal cycling of gltA-PCRs was performed the same as pan-*Bartonella* FRET-qPCR except removing the melting curve analysis and revising annealing temperature to 56°C. All the PCRs were run in duplicate.

### 2.5. Phylogenetic Analysis

**Figure 3:**
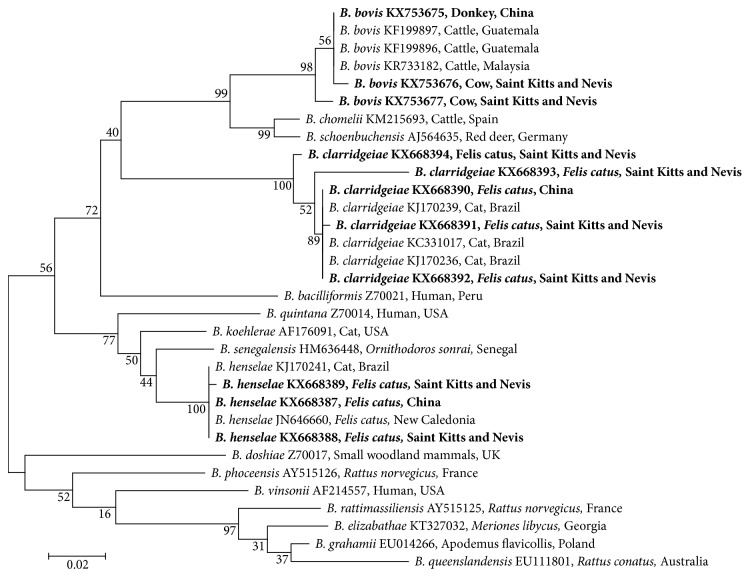
Molecular phylogenetic analysis of *Bartonella* spp. in this study. Distances and groupings of *Bartonella* spp. detected in this study (bold font) and reference *Bartonella* sequences from NCBI were determined by the maximum likelihood and Bayesian inference method with the MEGA version 6 software based on the *gltA* gene (439 bp). Bootstrap values were calculated using 500 replicates. Scale bar indicates a genetic distance of 0.02 nt substitutions per position.

The retrieved *Bartonella* sequences of the present study and the reference sequences from GenBank were aligned using the MEGA 6.0 software (Figure 3). Based on these alignments, phylogenetic trees were constructed by the maximum likelihood and Bayesian inference method using MEGA 6.0. Bootstrap values were calculated using 500 replicates.

## 3. Results

### 3.1. Testing of the Pan-*Bartonella* FRET-qPCR and Survey of Animals and Fleas for *Bartonella* spp. DNA

The probes we designed for the pan-*Bartonella* FRET-qPCR were highly conserved, while the primers only had between zero and four nucleotide mismatches with the 18 representative *Bartonella* spp. we used in the study (Figure 1). In contrast, the primers and probes had 11, 26, 27, 10, 17, 23, 22, and 29 mismatches with *A. phagocytophilum*, *A. marginale*, *Wolbachia*, *B. melitensis*, *B. chilestrain*, *R. felis*, *R. rickettsia*, and *C. burnetii*, respectively (Figure 1). The pan-*Bartonella* FRET-qPCR was positive with *B. henselae* DNA, but no products were obtained with DNAs from *Wolbachia*, *A. equi*, *A. marginale*, *B. melitensis*, *B. chilestrain*, *R. felis*, *R. rickettsia*, and *C. burnetii*. Using the gel-purified PCR products as quantitative standards [20], we determined the detection limit of the pan-*Bartonella* FRET-qPCR was two copies of the *Bartonella 16S rRNA* per reaction.

To confirm accuracy and reproducibility of real-time PCR, the intra-assay precision was determined in three repeats within one LightCycler run. Interassay variation was investigated in three different experimental runs, while the quantitative standards (1,000, 100, 10, and 1 copies/reaction) were diluted in 1 × T_10_E_0.1_, and with the diluents containing lambda DNA (New England Biolabs, Inc.) at the concentration of 1 ng/*μ*l. The calculation of test precision and test variability is based on the CP variation from the CP mean value. Test reproducibility for all investigated quantitative standards was lower in intratest experiments (<3.8%) and was higher in intertest experiments (<6.1%). The reproducibility dropped in lower copies of standards (1 and 10 copies per reaction) in comparison with the high copies of standards (100 copies and 1,000 copies).

Of the 310 cat blood samples we examined, 78 (25.2%) were positive in our pan-*Bartonella* FRET-qPCR. Cats from all the sites we studied were positive, mainly from Beijing (11.1%), Shanghai (5.6%), Guangdong (15.0%), Yangzhou (19.4%), and St. Kitts (39.7%) (Table 1). The 60 fleas collected from the Chinese cats were identified as *Ctenocephalides felis* based on their morphology, and 20.0% (12/60) were positive in the pan-*Bartonella* FRET-qPCR, while only one donkey (5.0%) from China was positive. Horses, sheep, and donkeys from St. Kitts were negative, but cattle from the island were commonly positive (54.8%) in the pan-*Bartonella* FRET-qPCR, although cattle from China were negative.

In general, the copies detected in the pan-*Bartonella* FRET-qPCR were considerably higher in the positive blood samples we collected (10^6.20^/ml whole blood). Fleas positive for *B. henselae* generally had low copy numbers, but those positive for *B. clarridgeiae* had considerably higher copy numbers (10^7.01^ vs. 10^2.88^).

### 3.2. Testing of the *gltA*-Based *Bartonella* PCR and Differentiation of the *Bartonella* spp. in the Survey Animals and Fleas

The *gltA*-based *Bartonella* PCR did not amplify the negative controls but did amplify the positive control *B. henselae* with an amplicon sequence identical to those for this species in GenBank. It also amplified all the test samples that were positive in the pan-*Bartonella* FRET-qPCR, indicating the tests had similar sensitivity. All of the cats that were positive in the pan-*Bartonella* FRET-qPCR were also positive in the *gltA*-based *Bartonella* PCR. Sequencing of the products of the *gltA*-based *Bartonella* PCR revealed *B. henselae* was most prevalent (64.1%; 50/78), followed by *B. clarridgeiae* (24.3%; 19/78) (Table 1). Both species were detected in cats from China and from St. Kitts. In the pan-*Bartonella* FRET-qPCR positive *C. felis* we collected from the Chinese cats, both *B. Bartonella henselae* (13.3%, 8/60) and *B. clarridgeiae* (6.7%, 4/60) were identified with the *gltA*-based *Bartonella* PCR. Each of the positive fleas was found on a cat positive for the same *Bartonella* sp.

Sequencing of amplicons from the *gltA*-based *Bartonella* PCR that were positive with the cattle from St. Kitts showed only *B. bovis* was present; this organism was also present in the one positive Chinese donkey (Table 1). The phylogenic comparison shows the identity or highly similar nucleotide sequences between *Bartonella* spp. identified in this work and those of the reference strains from GenBank (Table 2; Figure 3).

## 4. Discussion

As knowledge on the wide diversity and host ranges of *Bartonella* species increases, it becomes ever more important to develop reliable molecular assays that can detect all *Bartonella* species and thereby facilitate epidemiological and ecological studies. The pan-*Bartonella* FRET-qPCR we developed proved to be specific, not giving reactions with closely related organisms. This was expected as, by systematically aligning the sequences of 18 better-recognized *Bartonella* spp. in GenBank and other species, we were able to identify a highly conserved and specific region of the *16S rRNA* for the PCR (Figure 1). The pan-*Bartonella* FRET-qPCR was also very sensitive, detecting as few as two *Bartonella 16S rRNA* copies per reaction. Further advantages were that it was quantitative and relatively rapid to perform as gel electrophoresis was not needed to demonstrate amplicons.

Although the pan-*Bartonella* FRET-qPCR was very sensitive (2 copies per reaction) and specific, quantitative, and rapid (normally 1.5 hours including DNA extraction), because it was designed against a highly conserved region of the *16S rRNA*, it did not enable us to determine the *Bartonella* spp. in the samples. This was possible, however, with the *gltA*-based *Bartonella* PCR which was as accurate and specific as the pan-*Bartonella* FRET-qPCR in identifying *Bartonella* spp. in our study. Scola et al. first reported that, of the commonly used genetic targets for *Bartonella* identification, only *gltA* and *rpoB* sequences provide sufficient discriminatory power and interspecies diversity to allow discrimination of *Bartonella* spp. [33]. We therefore used *gltA* as the target of our PCR and selected our primers to ensure that, although they only amplified *Bartonella* spp., they covered a 439 bp hypervariable area of the gene that enabled differentiation of the recognized and poorly characterized *Bartonella* spp. recorded in GenBank (Figure 2). Our *gltA*-based PCR clearly differentiated the control *B. henselae* and identified three common species in our survey, *B. henselae*, *B. clarridgeiae*, and *B. bovis*, which were the species expected to be present in the animals surveyed.

Applying our pan-*Bartonella* FRET-qPCR to a number of convenience samples available enabled us to rapidly test a variety of samples from different geographic areas for *Bartonella*. We obtained positive results with all the samples tested, mainly mammalian whole blood, and vector insects (fleas) which comprise the most commonly used samples to date in epidemiological studies. Although the *gltA*-based *Bartonella* PCR was more time-consuming, requiring gel electrophoresis to reveal amplicons, it identified all the samples that were positive in the pan-*Bartonella* FRET-qPCR.

*Bartonella henselae* and *B. clarridgeiae* were the species most commonly identified by the *gltA*-based *Bartonella* PCR in our study. These are the organisms most commonly associated with cats and their fleas and have already been described to be prevalent in the West Indies [13] and China [40] where they are often associated with persistent subclinical bacteremia and transmitted by *C. felis* [2]. Although we found *B. bovis* at a high level in cattle from St. Kitts, we failed to identify the organism in cattle from China. We did, however, find *B. bovis* in a donkey in China, indicating the organism is present in the country. *Bartonella bovis* is a recently described species [41] that is generally the most prevalent of the three species that infect cattle, the others being *Bartonella schoenbuchensis* and *Bartonella chomelii*. The latter have mainly been described in Europe or in cattle originating from Europe [42], while *B. bovis* has been described from widely around the world with prevalences in cattle varying from 0% in Kenya and Japan [43] to up to 96% in the USA [44]. Although high levels of bacteremia can be found in cattle, persisting for up to seven months [45], most infections seem subclinical although cases of bovine endocarditis have been described [46]. There are little data on *B. bovis* in equids although horses seropositive against the organism have been described and experimental infections generally cause no clinical signs with no bacteremia and a low-grade and short-duration antibody response [47]. The vector of *B. bovis* has not been determined but is suspected to be a blood-sucking arthropod [48].

The specificity of the *16S rRNA*-based pan-*Bartonella* FRET-qPCR and the *gltA*-based *Bartonella* PCR we developed was based on the alignment of the available recognized *Bartonella* spp. sequences in GenBank. For completeness, the newly established PCRs should be tested on known positive control samples of DNA of all the recognized and poorly characterized *Bartonella* spp., but it is no simple matter for one laboratory to obtain all of these samples. Similarly, with the rapid growth in the recognized and suspected *Bartonella* species, it will be important for ongoing testing of the PCR to ensure it recognizes the newly recognized strains and species. Ideally, the established *Bartonella* PCRs in this work should be compared with other existing molecular approaches for their sensitivity and specificity and validated fully following the MIQE guidelines for publication of quantitative real-time PCR experiments [49].

In conclusion, in this report, we describe what we believe to be the first FRET-qPCR that specifically detects all the currently recognized *Bartonella* species. We also describe a *gltA*-based PCR that enables the differentiation of the recognized *Bartonella* spp. The pan-*Bartonella* FRET-qPCR enabled us to rapidly and quantitatively screen a variety of samples from a number of sources and different geographical areas for the presence of *Bartonella* spp., while the *gltA*-based PCR provided a convenient method to differentiate the species involved using only a single reaction.

## Figures and Tables

**Table 1:** Molecular prevalence of *Bartonella* in mammals.

Mammal	Source	Positive pan-*Bartonella* FRET-qPCR (*N*, %)	*Bartonella* sp. from *gltA*-based PCR	Average copies/ml blood, or per flea
Cat (*n* = 310)	Beijing	4/36, 11.1	4 *B. henselae*	10^5.60^
	Shanghai	2/36, 5.6	1 *B. henselae*	10^8.35^
			1 *B. clarridgeiae*	10^5.48^
	Guangdong	3/20, 15.0	3 *B. henselae*	10^4.60^
	Yangzhou	14/72, 19.4	11 *B. henselae*	10^6.36^
			3 *B. clarridgeiae*	10^7.86^
	St. Kitts	58/146, 39.7	38 *B. henselae*	10^6.33^
			20 *B. clarridgeiae*	10^6.65^

Cattle (*n* = 98)	St. Kitts	23/42, 54.8	23 *B. bovis*	10^6.03^
	Yangzhou	0/56, 0	—	

Horse (*n* = 24)	St. Kitts	0/24, 0	—	

Sheep (*n* = 30)	St. Kitts	0/30, 0	—	

Donkey (*n* = 24)	St. Kitts	0/4, 0	—	
	Hebei	1/20, 5.0	1 *B. bovis*	10^5.44^

*Ctenocephalides felis* (*n* = 60)	Yangzhou	12/60, 20.0	8 *B. henselae*	10^2.88^
			4 *B. clarridgeiae*	10^7.01^

**Table 2:** Comparison of isolates identified in this study and similar sequences in GenBank by BLAST.

Isolates identified in this study			Highly similar sequences in GenBank		
*Bartonella* spp.	Gene accession #	Source/origin	Gene accession #	Source/origin	Mismatches

*B. henselae*	KX668387	Cats from China	JN646660	Animals from New Caledonia	0/405
	KX668389	Cats from St. Kitts	KJ170241	Domestic cats from Brazil	1/397
	KX668388	Cats from St. Kitts	JN646660	Animals from New Caledonia	0/405

*B. clarridgeiae*	KX668391	Cats from St. Kitts	KJ170239	Domestic cats from Brazil	1/401
	KX668390	Cats from China	KJ170239	Domestic cats from Brazil	0/401
	KX668392	Cats from St. Kitts	KJ170236	Cats from animal shelters in Brazil	0/401
	KX668393	Cats from St. Kitts	KC331017	Cats from animal shelters in Brazil	14/401
	KX668394	Cats from St. Kitts	KC331017	Cats from animal shelters in Brazil	4/402

*B. bovis*	KX753675	Donkey from China	KR733182	Cattle from Malaysia	0/409
	KX753676	Cows from St. Kitts	KF199896	Cattle from Guatemala	0/409
	KX753677	Cows from St. Kitts	KF199897	Cattle from Guatemala	0/409

## Data Availability

Whole data including the nucleotide sequence used to support the findings of this study are included within the article.
